# Central obesity rather than BMI is associated with chronic pain: A cross-sectional analysis of NHANES

**DOI:** 10.1371/journal.pone.0337939

**Published:** 2025-12-04

**Authors:** Xu Yang, Xue Yang, Li Yang, Xuemei Dai, Lu Lin, Huaqu Gong, Yinghai Liu, Wei Wu

**Affiliations:** Department of Anesthesiology, The General Hospital of Western Theater Command, Chengdu, P.R.China; Instituto Nacional de Cardiologia Ignacio Chavez, MEXICO

## Abstract

**Background:**

Chronic pain is a prevalent and debilitating condition that poses a major public health burden. Most existing research on obesity and pain has focused on general obesity, typically assessed using body mass index (BMI). However, BMI fails to capture fat distribution and may not adequately reflect metabolic risks associated with pain. Central obesity, characterized by abdominal fat accumulation, has been increasingly recognized as a more relevant predictor of chronic disease, but its relationship with chronic pain remains underexplored in population-based studies.

**Methods:**

Data from 2,511 adults in the National Health and Nutrition Examination Survey (NHANES) were analyzed. Weighted logistic regression was used to assess the association between anthropometric indexes, including A Body Shape Index (ABSI), Waist Circumference (WC), Body Roundness Index (BRI), and BMI, and chronic pain. Subgroup and sensitivity analyses were conducted to test robustness. Restricted cubic spline (RCS) was applied to examine nonlinear relationships. Receiver operating characteristic (ROC) analysis compared the predictive performance of the anthropometric indicators.

**Results:**

Higher ABSI was significantly associated with increased odds of chronic pain, even after adjusting for a wide range of covariates including BMI (adjusted OR for highest vs. lowest quartile: 1.74; 95% CI: 1.16–2.59; *P* = 0.010). In contrast, BRI, WC, and BMI were not significantly associated with chronic pain. RCS analysis indicated a linear relationship between ABSI and chronic pain. ROC analysis showed that central obesity indicators (ABSI, BRI, and WC) had better discriminative ability than BMI. Findings were consistent across subgroup and sensitivity analyses.

**Conclusion:**

Central obesity, as measured by ABSI, is significantly associated with chronic pain, independent of BMI and other risk factors. These findings highlight the importance of incorporating central obesity indicators into public health and clinical strategies for chronic pain prevention and management.

## 1. Introduction

Chronic pain is typically defined as “pain that persists for at least three months on most days” [1]. Specifically, chronic non-cancer pain refers to pain that lasts longer than three months and is unrelated to tumors [2]. It is a complex pathological state involving the interaction of neurobiological, psychological, and social factors [3]. Chronic pain is a major global public health issue, affecting approximately 30% of the general population, significantly reducing patients’ quality of life and occupational function, and leading to additional medical costs [4]. In specific populations, prevalence is notably high among children and adolescents, with approximately 25% of children reporting chronic and recurrent pain, of which 8% experience severe and frequent pain; long-term effects may lead to lifelong use of analgesic medications [5]. The incidence of chronic pain is rising globally due to population aging and increased prevalence of neurological diseases (such as diabetes, cancer, autoimmune diseases, trauma, and infections), affecting approximately 7%–10% of the global population [6].

Obesity leads to abnormal adipose tissue function, releasing pro-inflammatory cytokines (such as tumor necrosis factor-α and interleukin-6) and adipokines (such as leptin and adiponectin). These molecules enhance peripheral pain pathways, leading to pain sensitization (e.g., in OA patients), and trigger systemic pain by upregulating high-sensitivity C-reactive protein (hsCRP) [7–9]. Additionally, glial cells (such as microglia and astrocytes) are activated by obesity-induced inflammation, releasing pro-inflammatory cytokines, thereby enhancing pain transmission and chronicization in the central nervous system. Increased visceral fat is often associated with metabolic syndrome (e.g., insulin resistance), which independently of BMI (body mass index) exerts a specific influence on OA-related pain by amplifying pain through inflammatory and immune pathways [10]. Visceral fat drives OA pain through both local (perarticular fat pads) and systemic inflammation, and adipose-derived factors (such as leptin) can directly promote joint degeneration and pain sensitization [11,12]. Chronic pain (such as OA) may lead to muscle atrophy and fat infiltration, further exacerbating muscle dysfunction and pain [13,14]. For example, in patients with chronic back pain, increased intermuscular fat tissue (InterMAT) is associated with pain severity [14]. Therefore, obesity increases the incidence of pain and may exacerbate its severity and persistence through mechanisms such as inflammation, neuroendocrine dysfunction, pain sensitization, and metabolic disorders [7,15,16]. These mechanisms are not independent but intertwined (e.g., inflammation triggers sensitization), leading to reduced pain management efficacy in obese individuals.

Traditional indicators like BMI and Waist Circumference (WC) have limitations when assessing obesity-related health risks: BMI cannot distinguish between muscle and fat distribution, while WC, though it reflects abdominal obesity, does not account for height, leading to weaker and less stable associations with chronic pain [17]. This highlights the need for more effective indicators to capture the heterogeneous effects of obesity, particularly those related to pain mechanisms associated with fat distribution.

A Body Shape Index (ABSI), as an emerging indicator, optimizes the quantification of body shape through mathematical formulas (e.g., ABSI adjusts for the influence of waist circumference on height), better reflecting the characteristics of central obesity [18]. Numerous studies have shown that ABSI outperforms traditional indicators in predicting health outcomes. For example, in an analysis assessing the risk of cognitive impairment, ABSI demonstrated higher diagnostic accuracy [10]. When predicting mortality risk, ABSI exhibited stronger predictive capability, particularly for outcomes related to central obesity [19]. Additionally, ABSI demonstrated higher comprehensive value in predicting the risk of specific diseases, especially cardiovascular and metabolic diseases [20,21]. This suggests that ABSI can more sensitively capture the pathological effects of visceral fat, which is considered an important driver of chronic pain [22].

## 2. Methods

### 2.1 Study design and participants

**Fig 1:**
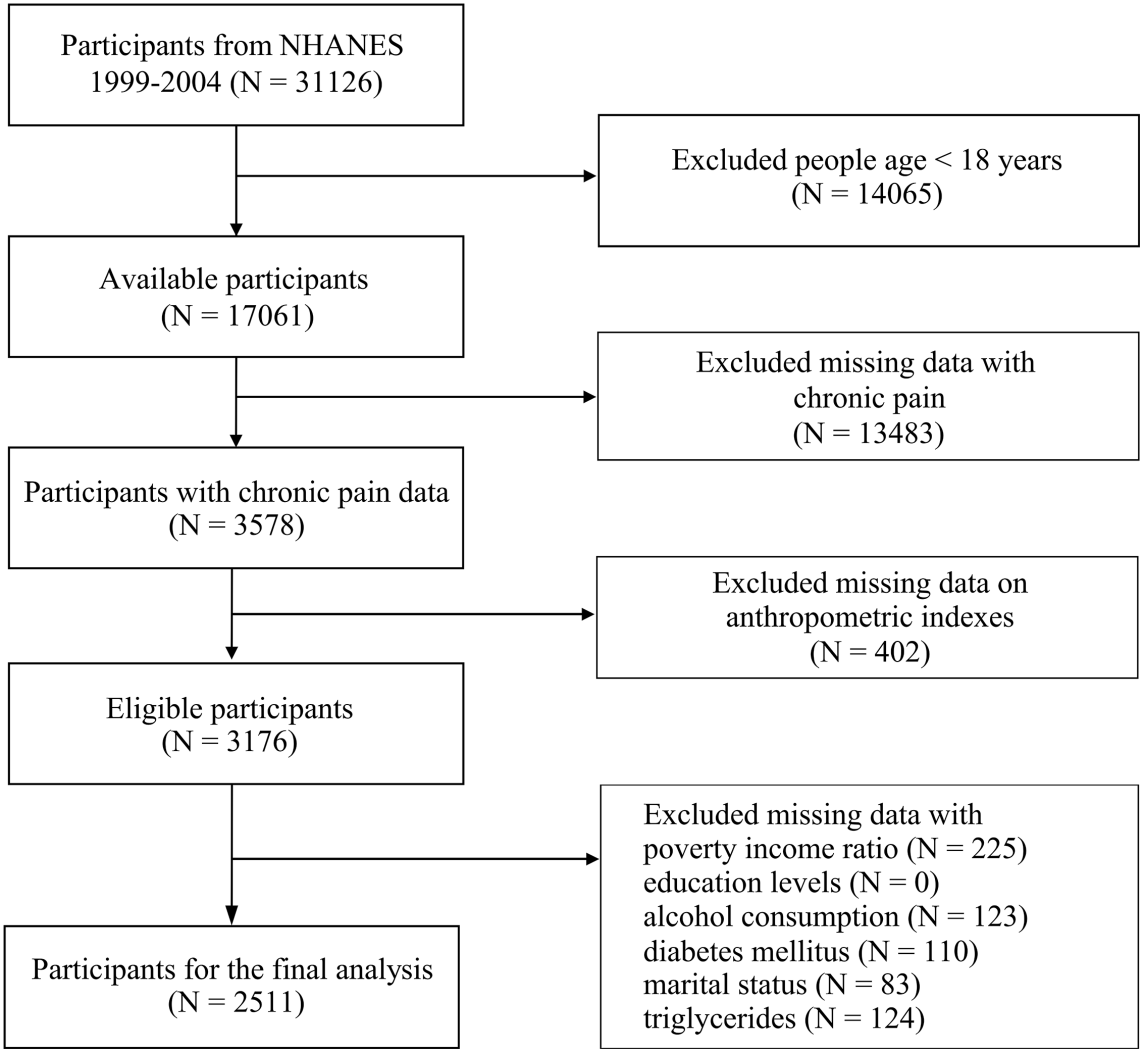
Flowchart of procedures for participants selection and inclusion.

NHANES, a nationally representative program conducted in the United States. NHANES employs a complex, multistage probability sampling design, which allows the weighted data to represent the non-institutionalized civilian U.S. population. We selected data from the 1999–2000, 2001–2002, and 2003–2004 cycles because information on chronic pain was only available during these specific periods. Fig 1 shows the inclusion and exclusion criteria. After applying appropriate sample weights, these participants represent an estimated 42,445,198 individuals in the U.S. population.

### 2.2 Ethics statement

All participants provided written informed consent. The NHANES study protocols were reviewed and approved by the Ethics Review Board (ERB) of the National Center for Health Statistics (NCHS). The 1999−2004 NHANES cycle was conducted under the Continuation of Protocol #98−12.

### 2.3 Assessment of anthropometric indexes

Anthropometric measurements were conducted by trained professionals at mobile examination centers (MEC). WC was measured directly and did not require calculation. BMI, the most commonly used indicator of general obesity, was calculated using the following formula: BMI = Weight (kg)/Height (m)^2^.

ABSI and Body Roundness Index (BRI) are two newer indicators developed to reflect central obesity, with each emphasizing different aspects of body shape and fat distribution. The formula for ABSI is:


$$\text{ABSI}=\frac{\text{WC}}{{\text{BMI}}^{\text{2}/\text{3}}\times {\text{height}}^{\text{1}/\text{2}}}$$

The formula for BRI is:


$$\text{B}\text{RI}=\text{364}.\text{2}-\text{365}.\text{5}\times \sqrt{\text{1}-({\frac{\text{WC}}{2\pi })}^{\text{2}}/{(\text{0}.\text{5}\times \text{height})}^{\text{2}}}$$

### 2.4 Assessment of chronic pain

In NHANES 1999–2004, the duration of pain was assessed using the variable MPQ110, which appeared consistently across the Miscellaneous Pain questionnaires, mpq (1999–2000), mpq_b (2001–2002), and mpq_c (2003–2004). This item asked participants: “For how long have you experienced this pain?” The available response options were: (1) Don’t know; (2) Refused; (3) Less than a month; (4) At least 1 month but less than 3 months; (5) At least 3 months but less than 1 year; (6) Greater than 1 year. In this study, chronic pain was defined as pain lasting three months or longer, based on the participant’s response to this item. In sensitivity analyses, an alternative definition was applied, classifying chronic pain as lasting more than one year.

### 2.5 Definition of covariates

The selected covariates included commonly used demographic and epidemiological variables relevant to chronic pain and obesity research: age, sex, race/ethnicity, poverty income ratio (PIR), body mass index (BMI), marital status, education levels, smoking status, alcohol consumption, hyperlipidemia, hypertension, and diabetes mellitus (defined in S1 Table). In addition, since ABSI, BRI, WC, and BMI are all closely related to body fat, triglycerides were also included as an additional covariate to control for metabolic factors. The detailed definitions and categorizations of all covariates are presented in Table 1.

**Table 1:** Descriptive characteristics of the study population stratified by chronic pain.

Characteristic	Total	Non-Chronic Pain Group	Chronic Pain Group	*P*-values
	(N = 2511)	(N = 1019)	(N = 1492)	
**A Body Shape Index**	8.07 ± 0.02	7.98 ± 0.02	8.14 ± 0.02	**< 0.001**
**Body Roundness Index**	5.20 ± 0.07	5.01 ± 0.10	5.35 ± 0.07	**< 0.001**
**Waist Circumference (m)**	0.98 ± 0.00	0.97 ± 0.01	0.99 ± 0.00	**0.006**
**Body Mass Index (kg/m**^**2**^)	28.94 ± 0.21	28.86 ± 0.34	29.00 ± 0.21	0.666
**Triglycerides (mmol/L)**	1.73 ± 0.05	1.68 ± 0.08	1.78 ± 0.06	0.357
**Age, n (%)**				**< 0.001**
18-29	327 (14.41)	190 (58.57)	137 (41.43)	
30-44	708 (33.86)	321 (46.48)	387 (53.52)	
45-59	694 (32.76)	273 (39.49)	421 (60.51)	
>=60	782 (18.97)	235 (29.01)	547 (70.99)	
**Sex, n (%)**				0.348
Female	1336 (53.71)	527 (41.24)	809 (58.76)	
Male	1175 (46.29)	492 (44.21)	683 (55.79)	
**Race/Ethnicity, n (%)**				0.065
Non‐Hispanic white	1497 (78.23)	582 (41.70)	915 (58.30)	
Non‐Hispanic black	420 (8.36)	172 (42.50)	248 (57.50)	
Hispanic	511 (9.48)	236 (51.02)	275 (48.98)	
other race	83 (3.94)	29 (40.93)	54 (59.07)	
**Body Mass Index, n (%)**				0.455
Underweight/Normal	717 (30.17)	316 (44.94)	401 (55.06)	
Overweight	869 (33.95)	357 (42.08)	512 (57.92)	
Obese	925 (35.88)	346 (41.17)	579 (58.83)	
**Marital status, n (%)**				**< 0.001**
Married/Living with Partner	1585 (66.73)	632 (40.86)	953 (59.14)	
Never married	333 (13.20)	185 (58.27)	148 (41.73)	
Widowed/Divorced/Separated	593 (20.06)	202 (38.18)	391 (61.82)	
**Poverty income ratio, n (%)**				**0.010**
< 1.3	744 (23.31)	265 (36.70)	479 (63.30)	
1.3-3.5	931 (35.46)	372 (40.94)	559 (59.06)	
> 3.5	836 (41.24)	382 (47.40)	454 (52.60)	
**Education levels, n (%)**				**0.002**
Below high school	294 (5.10)	112 (38.62)	182 (61.38)	
High school	1062 (42.15)	395 (38.46)	667 (61.54)	
Above high school	1155 (52.75)	512 (46.33)	643 (53.67)	
**Alcohol consumption, n (%)**				**0.004**
Never	305 (10.85)	129 (46.65)	176 (53.35)	
Former	584 (19.98)	189 (32.68)	395 (67.32)	
Mild	773 (31.92)	328 (45.65)	445 (54.35)	
Moderate	359 (15.64)	160 (44.15)	199 (55.85)	
Heavy	490 (21.61)	213 (44.21)	277 (55.79)	
**Smoking status, n (%)**				**< 0.001**
Never	1112 (43.62)	500 (48.75)	612 (51.25)	
Former	707 (26.60)	271 (41.37)	436 (58.63)	
Now	692 (29.78)	248 (34.76)	444 (65.24)	
**Hyperlipidemia, n (%)**				0.110
No	611 (25.06)	280 (46.10)	331 (53.90)	
Yes	1900 (74.94)	739 (41.46)	1161 (58.54)	
**Hypertension, n (%)**				**< 0.001**
No	1363 (60.45)	624 (46.82)	739 (53.18)	
Yes	1148 (39.55)	395 (36.19)	753 (63.81)	
**Diabetes mellitus, n (%)**				**< 0.001**
No	2047 (85.70)	876 (44.42)	1171 (55.58)	
Prediabetes	96 (3.30)	29 (27.66)	67 (72.34)	
DM	368 (11.00)	114 (33.05)	254 (66.95)	

*To facilitate data presentation and interpretation, A Body Shape Index (ABSI) values were multiplied by 100 due to their relatively small magnitude.

### 2.6 Statistical analyses

All statistical analyses were performed using R Studio (version 4.3.1). Based on NHANES recommendations and the variables used in this study, MEC sample weights were applied. Specifically, for data from 1999–2002, the weight was calculated as 2/3*WTMEC4YR, and for 2003–2004, it was 1/3*WTMEC2YR. For weighted logistic regression analyses, anthropometric indexes were analyzed both as continuous variables and as categorical variables based on quartile stratifications. The specific cutoff points for categorization are provided in S2 Table. All variance inflation factors (VIF) were below 2.2, indicating no significant multicollinearity. The linearity assumption for logistic regression (logit(P)) was verified. Subgroup analyses were conducted to assess the consistency of associations. Two sensitivity analyses were performed: one without applying sample weights, and another using an alternative definition of chronic pain as lasting more than one year. Restricted cubic spline (RCS) models were used to explore potential non-linear relationships. Receiver operating characteristic (ROC) analysis was employed to compare the predictive performance.

## 3. Results

### 3.1 Descriptive characteristics

Of the 2,511 participants aged ≥18 years included in the final sample, 1,492 reported experiencing chronic pain. As shown in Table 1, ABSI, BRI, and WC were significantly higher in the chronic pain group compared to the non-chronic pain group. Specifically, the mean ABSI was 8.14 ± 0.02 vs. 7.98 ± 0.02 (*P* < 0.001), BRI was 5.35 ± 0.07 vs. 5.01 ± 0.10 (*P* < 0.001), and WC was 0.99 ± 0.00 m vs. 0.97 ± 0.01 m (*P* = 0.006). In contrast, BMI did not differ significantly between groups (29.00 ± 0.21 vs. 28.86 ± 0.34 kg/m²; *P* = 0.666), suggesting that BMI may be less sensitive in distinguishing individuals with chronic pain compared to central obesity indicators. In addition, the prevalence of chronic pain was significantly higher among older adults, individuals who were widowed/divorced/separated, those with a PIR < 1.3, lower education levels, smokers, and those with hypertension or diabetes mellitus. No significant differences in chronic pain prevalence were observed by sex, race/ethnicity, BMI categories, or hyperlipidemia status.

### 3.2 Association between anthropometric indexes and chronic pain

Table 2 presents the adjusted associations between anthropometric indexes and chronic pain. BMI, whether analyzed as a continuous or categorical variable, showed no significant association with chronic pain in any of the models. In the unadjusted model, higher ABSI, BRI, and WC were all significantly associated with increased odds of chronic pain. However, in adjust model 1, only ABSI remained significantly associated. Specifically, when treated as a continuous variable, ABSI had an OR of 1.77 (95% CI: 1.42–2.20; *P *< 0.001). When categorized into quartiles, compared to Q1, the ORs were 1.42 (1.07–1.88; *P *= 0.016) for Q2, 1.72 (1.29–2.29; *P *< 0.001) for Q3, and 2.03 (1.44–2.84; *P *< 0.001) for Q4. In adjust model 2, ABSI still showed a significant association with chronic pain. The OR for ABSI as a continuous variable was 1.58 (1.21–2.07; *P *= 0.002). When analyzed as a categorical variable, Q3 and Q4 remained significantly associated with chronic pain, with ORs of 1.60 (1.13–2.26; *P *= 0.011) and 1.74 (1.16–2.59; *P *= 0.010), respectively. This effect size suggests a small-to-moderate clinical association. The *P* for trend was statistically significant across all models for ABSI.

**Table 2:** Adjusted association of anthropometric indexes with chronic pain.

Exposure	Unadjusted model	Adjust 1	Adjust 2
	Odds ratio (95% confidence interval) associated with chronic pain		
ABSI (continuous)*	1.99 (1.66, 2.40); **< 0.001**	1.77 (1.42, 2.20); **< 0.001**	1.58 (1.21, 2.07); **0.002**
**Quartile of ABSI**			
Q1	1 (Ref)	1 (Ref)	1 (Ref)
Q2	1.41 (1.08, 1.85); **0.013**	1.42 (1.07, 1.88); **0.016**	1.36 (0.99, 1.86); 0.057
Q3	1.83 (1.38, 2.42); **< 0.001**	1.72 (1.29, 2.29); **< 0.001**	1.60 (1.13, 2.26); **0.011**
Q4	2.46 (1.87, 3.25); **< 0.001**	2.03 (1.44, 2.84); **< 0.001**	1.74 (1.16, 2.59); **0.010**
*P* for trend	**< 0.001**	**< 0.001**	**0.007**
**BRI (continuous)**	1.07 (1.02, 1.11); **0.002**	1.03 (0.99, 1.08); 0.171	1.03 (0.97, 1.10); 0.294
**Quartile of BRI**			
Q1	1 (Ref)	1 (Ref)	1 (Ref)
Q2	1.10 (0.83, 1.46); **0.048**	0.97 (0.72, 1.31); 0.837	1.04 (0.70, 1.54); 0.834
Q3	1.61 (1.19, 2.18); **0.003**	1.30 (0.92, 1.85); 0.136	1.43 (0.82, 2.49); 0.193
Q4	1.43 (1.14, 1.79); **0.003**	1.16 (0.87, 1.53); 0.299	1.32 (0.75, 2.34); 0.315
*P* for trend	**< 0.001**	0.150	0.255
**WC (continuous)**	2.31 (1.24, 4.31); **0.010**	1.63 (0.80, 3.35); 0.175	2.20 (0.73, 6.60); 0.149
**Quartile of WC**			
Q1	1 (Ref)	1 (Ref)	1 (Ref)
Q2	1.14 (0.83, 1.57); 0.409	1.04 (0.73, 1.48); 0.840	1.10 (0.70, 1.72); 0.674
Q3	1.35 (1.01, 1.81); **0.042**	1.13 (0.82, 1.57); 0.439	1.24 (0.70, 2.19); 0.429
Q4	1.40 (1.09, 1.80); **0.010**	1.20 (0.87, 1.65); 0.263	1.28 (0.66, 2.49); 0.438
*P* for trend	**0.006**	0.219	0.408
**BMI (continuous)**	1.00 (0.99, 1.02); 0.669	1.00 (0.98, 1.01); 0.856	0.99 (0.98, 1.01); 0.096
**Quartile of BMI**			
< 25	1 (Ref)	1 (Ref)	1 (Ref)
25-30	1.12 (0.86, 1.46); 0.380	1.01 (0.75, 1.37); 0.921	1.04 (0.76, 1.42); 0.794
≥ 30	1.17 (0.90, 1.51); 0.235	1.06 (0.80, 1.41); 0.692	1.02 (0.77, 1.36); 0.891
*P* for trend	0.241	0.683	0.901

Unadjusted model: non-adjusted model.

Adjust 1: Adjust for age, sex, race.

Adjust 2: Adjust for age, sex, race, body mass index, poverty income ratio, education levels, marital status, smoking status, alcohol consumption, hyperlipidemia, hypertension, diabetes mellitus and triglycerides.

*To facilitate data presentation and interpretation, ABSI values were multiplied by 100 due to their relatively small magnitude.

**Abbreviations**: ABSI, A Body Shape Index; BRI, body roundness index; WC, Waist Circumference; BMI, Body Mass Index; CI, confidence interval.

These results suggest that ABSI is independently and robustly associated with chronic pain, even after controlling for multiple confounders, whereas BRI, WC, and BMI do not maintain significant associations in fully adjusted models. ABSI may therefore serve as a more sensitive and reliable anthropometric indicator in identifying individuals at risk for chronic pain.

### 3.3 Subgroup and sensitivity analyses

**Fig 2:**
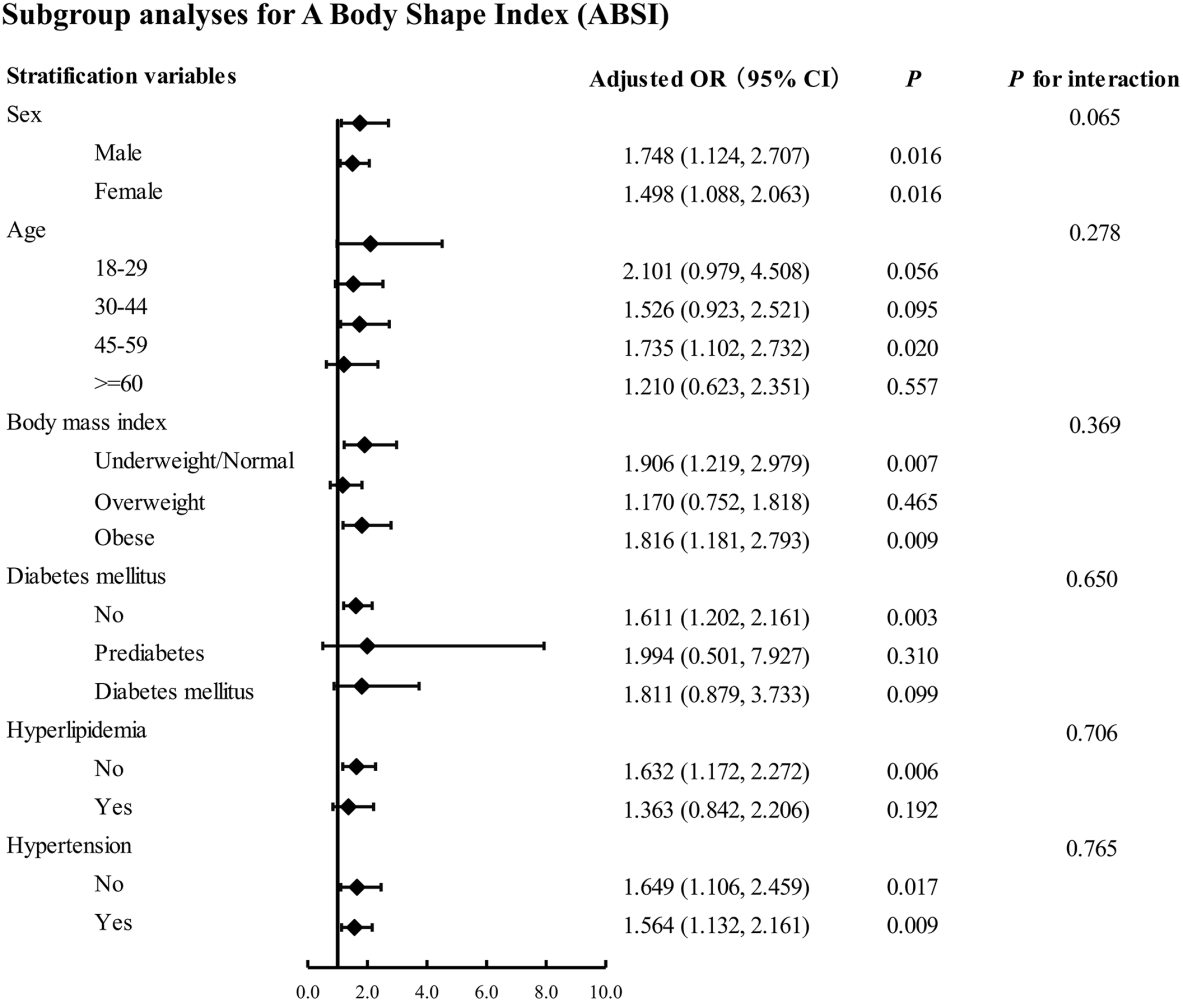
Adjusted association of ABSI with chronic pain for subgroup analyses. All analyses were adjusted for age, sex, race, body mass index, poverty income ratio, education levels, marital status, smoking status, alcohol consumption, hyperlipidemia, hypertension, diabetes mellitus and triglycerides, but not for the specific stratification variables of interest.

Subgroup analyses were conducted to assess whether the association between ABSI and chronic pain varied across population subgroups, i.e., to explore potential effect modification. As shown in Fig 2, the interaction *P*-values were greater than 0.05 for all subgroup comparisons. Although the association between ABSI and chronic pain did not differ significantly by sex (P for interaction = 0.065), we observed a slightly higher effect estimate in males (OR = 1.748, 95% CI: 1.124–2.707) than in females (OR = 1.498, 95% CI: 1.088–2.063). This numerical difference may warrant further investigation in future studies but should be interpreted with caution given the lack of a statistically significant interaction.

In S3 Table, unweighted logistic regression models were used as a sensitivity analysis. The results were highly consistent with those from the weighted analysis. In Model 2, ABSI remained significantly associated with chronic pain. Similarly, in S4 Table, chronic pain was redefined as pain lasting more than one year. Under this alternative outcome definition, ABSI continued to show a significant association with chronic pain, aligning with the primary findings.

These results support the robustness and stability of the association between ABSI and chronic pain, regardless of weighting methods, chronic pain definitions, or population subgroups.

### 3.4 Nonlinear relationships explore

**Fig 3:**
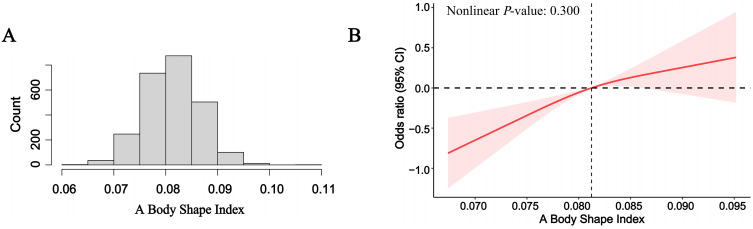
The distribution of ABSI (A). The full-adjusted relationship between ABSI with chronic pain using Restricted Cubic Spline (B). The solid line represents the fitted nonlinear curve. The area adjacent to the solid line represents the 95% confidence interval. Abbreviations: CI, confidence interval; ABSI, A Body Shape Index.

As shown in Fig 3A, the distribution of ABSI approximated normality. To investigate potential nonlinear associations between ABSI and chronic pain, RCS regression was applied. As illustrated in Fig 3B, the model using 3 knots yielded a nonlinear *P*-value of 0.300, indicating no significant evidence of a nonlinear relationship. To further assess the robustness of this finding, additional models were tested using knots ranging from 3 to 8. The corresponding *P*-values for nonlinearity were greater than 0.05 in all cases except when using 5 knots (S5 Table).

### 3.5 Predictive value of anthropometric indexes for chronic pain

**Fig 4:**
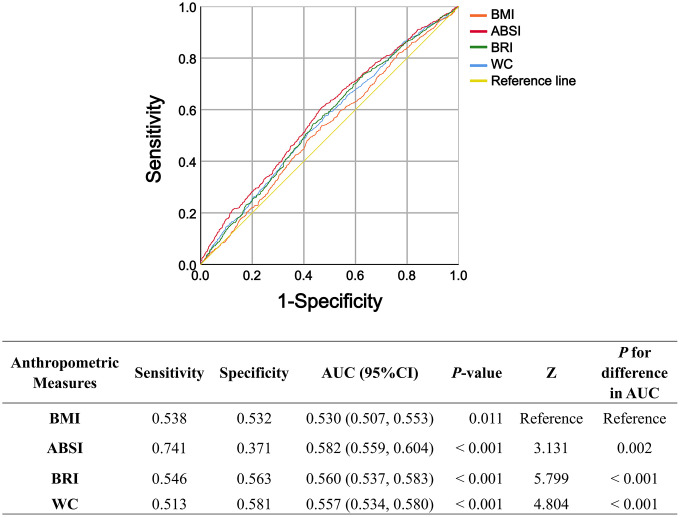
The Receiver Operating Characteristic (ROC) curves for the prediction of chronic pain. The Z-test of the area under the ROC curve (AUC) is used to assess the significant difference in predictive performance between the prediction models. Abbreviations: ABSI, A Body Shape Index; BRI, body roundness index; WC, Waist Circumference; BMI, Body Mass Index; CI, confidence interval.

To further assess the predictive performance of central obesity indicators relative to the conventional general obesity measure (BMI), ROC curve analyses were conducted. As illustrated in Fig 4, the area under the curve (AUC) for BMI was 0.530 (95% CI: 0.507–0.553), indicating limited discriminatory ability. In contrast, AUCs for ABSI, BRI, and WC were 0.582 (95% CI: 0.559–0.604; *P *< 0.001), 0.560 (95% CI: 0.537–0.583; *P *< 0.001), and 0.557 (95% CI: 0.534–0.580; *P* < 0.001), respectively. Z-tests comparing AUCs revealed that ABSI, BRI, and WC each had significantly greater discriminative power for chronic pain compared to BMI. These findings reinforce the superior predictive value of central obesity indicators, particularly ABSI, in identifying individuals at higher risk of chronic pain.

## 4. Discussion

We found that anthropometric indicators reflecting central obesity, namely ABSI, BRI, and WC, demonstrated significantly better predictive performance for chronic pain compared to the conventional indicator of general obesity, BMI. Among them, ABSI exhibited the strongest and most consistent association with chronic pain, remaining significant even after adjustment for a wide range of covariates, including BMI. In contrast, BMI showed no significant association with chronic pain across any regression model. These findings underscore the potential advantage of central obesity measures, particularly ABSI, in identifying individuals at higher risk for chronic pain.

Previous studies have widely reported associations between general obesity and pain in specific populations and pain types, with most relying exclusively on BMI as the measure of adiposity [23,24]. While longitudinal data have demonstrated that higher BMI can exacerbate lower back pain [23], our study shows that central obesity indicators, particularly ABSI, outperform BMI in predicting chronic pain as a generalized and persistent pain condition. Unlike traditional measures, ABSI can distinguish differences in fat distribution and muscle mass, which may explain why other indices such as BMI, WC, and BRI lost their significance after adjustment for covariates, while ABSI remained independently associated [18,19]. Furthermore, ABSI is linked to inflammation and metabolic dysfunction, both of which play a crucial role in the pathophysiology of chronic pain [25]. This finding aligns with the study by Kang et al., which demonstrated that ABSI was significantly associated with depressive symptoms in a Korean population [26]. Notably, prior meta-analyses examining waist circumference and waist-to-hip ratio reported only weak correlations with pain intensity (r = 0.09–0.11) [17], whereas our study, by applying ABSI as a more precise indicator, revealed a stronger dose–response relationship between central obesity and chronic pain. These results support emerging theories suggesting that adipose tissue dysfunction may contribute to chronic pain through systemic and local inflammation, immune dysregulation, and the production of pro-inflammatory cytokines [27]. ABSI may be better positioned than other anthropometric measures to capture these underlying pathophysiological processes.

The association between central obesity and chronic pain likely involves complex neuroendocrine and neuroimmune regulatory processes. Multiple regulatory factors within neuroendocrine pathways, including leptin, ghrelin, and galanin, are involved in both obesity and pain modulation. These factors interact with other neuropeptides and hormonal systems to influence nociceptive regulation [28]. In addition, obesity-related metabolic syndrome may intensify pain experiences in conditions such as osteoarthritis through both peripheral and central sensitization mechanisms. Visceral hypersensitivity, observed in disorders like irritable bowel syndrome (IBS), may also involve gut microbiota modulation of central pain pathways [27,29]. Recent studies have further shown that spinal microglia promote central sensitization via P2Y12 receptor signaling, offering new therapeutic targets for obesity-related chronic visceral pain [30]. Together, these findings underscore a multifaceted interplay of neuroendocrine dysregulation, immune activation, and central nervous system plasticity in mediating the link between obesity and chronic pain [9,31].

This study has several notable strengths. First, the consistent findings across multiple analytic approaches, including weighted logistic regression, subgroup and sensitivity analyses, restricted cubic spline models, and ROC analysis, underscore the robustness and reliability of the results. Second, the identification of ABSI as a superior marker for chronic pain risk advances the field by highlighting the limitations of BMI, which does not adequately account for fat distribution or metabolic activity. As this is a cross-sectional study, causal relationships between central obesity and chronic pain cannot be established. In addition, we were unable to differentiate pain subtypes due to limitations in the NHANES data. Mental health factors, such as depression and sleep disorders, were not included because the necessary variables were not available in the 1999–2004 NHANES cycles. Finally, although ABSI, BRI, and WC demonstrated statistically greater discriminative ability than BMI, the overall predictive performance of all these anthropometric indicators was limited, and their clinical utility for identifying chronic pain risk remains modest.

It should be noted that NHANES includes only non-institutionalized, community-dwelling individuals, excluding those in long-term care or other institutional settings. This limits the generalizability of our findings, as institutionalized populations often have a higher prevalence of chronic pain due to greater comorbidities and functional decline. Future studies should consider linkage with institutional health data or conduct targeted investigations in such populations to validate and extend current findings.

Our analysis is based on NHANES data collected between 1999 and 2004. Since then, important changes have occurred in opioid prescribing practices, cannabis regulation, and working conditions, which may influence the current patterns of chronic pain. Therefore, caution is needed when generalizing our findings to contemporary populations. Nonetheless, the association between central obesity and chronic pain is likely to persist, given that it is driven by relatively stable biological mechanisms such as inflammation and metabolic dysfunction. Future studies using more recent and publicly available datasets are needed to confirm whether this association remains consistent under current social and clinical conditions.

Despite these limitations, the study holds important public health implications. Chronic pain is a growing burden globally, and obesity is one of its modifiable risk factors. Our findings suggest that incorporating central obesity indicators, especially ABSI, into screening and prevention strategies may enhance early identification of individuals at high risk for chronic pain, beyond what BMI alone can offer. This approach could help refine risk stratification and enable more targeted health interventions. These insights support a shift toward more nuanced anthropometric assessments in both clinical practice and epidemiological research, ultimately contributing to more effective chronic pain management at the population level.

## 5. Conclusions

In summary, this nationally representative cross-sectional study reveals that central obesity, particularly as measured by ABSI, is significantly and independently associated with chronic pain among U.S. adults. Unlike traditional indicators such as BMI, which showed no significant association, ABSI exhibited a robust and consistent association with chronic pain, even after adjusting for BMI and other key confounders. These findings highlight the limitations of relying solely on BMI for obesity-related pain risk assessment and underscore the importance of incorporating central obesity measures into epidemiological research and clinical practice. Targeting abdominal adiposity may offer more effective strategies for chronic pain prevention and management.

## Supporting information

S1 Table The detailed overview of how we obtained information on hypertension, hyperlipidemia, and diabetes. (DOCX)

S2 Table Stratification details of anthropometric indexes (a body shape index, body roundness index, waist circumference). (DOCX)

S3 Table Sensitivity analysis of the relationship between A Body Shape Index and chronic pain was conducted without applying weighting. Unadjusted model: non-adjusted model. Adjust 1: Adjust for age, sex, race. Adjust 2: Adjust for age, sex, race, body mass index, poverty income ratio, education levels, marital status, smoking status, alcohol consumption, hyperlipidemia, hypertension, diabetes mellitus and triglycerides. * To facilitate data presentation and interpretation, ABSI values were multiplied by 100 due to their relatively small magnitude. Abbreviations: ABSI, A Body Shape Index; CI, confidence interval.
(DOCX)

S4 Table Sensitivity analysis was conducted by redefining chronic pain as pain lasting more than one year. Unadjusted model: non-adjusted model. Adjust 1: Adjust for age, sex, race. Adjust 2: Adjust for age, sex, race, body mass index, poverty income ratio, education levels, marital status, smoking status, alcohol consumption, hyperlipidemia, hypertension, diabetes mellitus and triglycerides. * To facilitate data presentation and interpretation, ABSI values were multiplied by 100 due to their relatively small magnitude. Abbreviations: ABSI, A Body Shape Index; CI, confidence interval.
(DOCX)

S5 Table Nonlinear *P*-values of ABSI and chronic pain at different knots. Abbreviations: ABSI, A Body Shape Index.
(DOCX)
