# Supplementary material for: Central obesity rather than BMI is associated with chronic pain: A cross-sectional analysis of NHANES
Source: PLoS One. 2025 Dec 4;20(12):e0337939. doi: 10.1371/journal.pone.0337939 (PMC12677471; doi:10.1371/journal.pone.0337939)
Supplement: S2 Table — (DOCX) [file pone.0337939.s002.docx]

| **Variables** | **Division details** | **Corresponding range (mg)** | **Frequency** | **Percentage** |
| --- | --- | --- | --- | --- |
| **A body shape index** | Q1 | [0.063, 0.078] | 668 | 26.6% |
|  | Q2 | (0.078, 0.081] | 531 | 21.15% |
|  | Q3 | (0.081, 0.085] | 695 | 27.68% |
|  | Q4 | (0.085, 0.109] | 617 | 24.57% |
| **Body roundness index** | Q1 | [1.361, 3.769] | 628 | 25.01% |
|  | Q2 | (3.769, 5.035] | 627 | 24.97% |
|  | Q3 | (5.035, 6.542] | 627 | 24.97% |
|  | Q4 | (6.542, 18.14] | 629 | 25.05% |
| **Waist circumference** | Q1 | [0.618, 0.878] | 630 | 25.09% |
|  | Q2 | (0.878, 0.98] | 627 | 24.97% |
|  | Q3 | (0.98, 1.09] | 635 | 25.29% |
|  | Q4 | (1.09, 1.73] | 619 | 24.65% |

**Table S2**. Stratification details of anthropometric indexes (a body shape index, body roundness index, waist circumference).
