# Supplementary material for: Central obesity rather than BMI is associated with chronic pain: A cross-sectional analysis of NHANES
Source: PLoS One. 2025 Dec 4;20(12):e0337939. doi: 10.1371/journal.pone.0337939 (PMC12677471; doi:10.1371/journal.pone.0337939)
Supplement: S3 Table — Unadjusted model: non-adjusted model. Adjust 1: Adjust for age, sex, race. Adjust 2: Adjust for age, sex, race, body mass index, poverty income ratio, education levels, marital status, smoking status, alcohol consumption, hyperlipidemia, hypertension, diabetes mellitus and triglycerides. * To facilitate data presentation and interpretation, ABSI values were multiplied by 100 due to their relatively small magnitude. Abbreviations: ABSI, A Body Shape Index; CI, confidence interval. (DOCX) [file pone.0337939.s003.docx]

**Table S3*.*** Sensitivity analysis of the relationship between A Body Shape Index and chronic pain was conducted without applying weighting.

| **Exposure** | **Unadjusted model** | **Adjust 1** | **Adjust 2** |
| --- | --- | --- | --- |
|  | Odds ratio (95% confidence interval) associated with chronic pain | | |
| **ABSI (continuous)*** | 1.78 (1.53, 2.07); **< 0.001** | 1.49 (1.25, 1.79); **< 0.001** | 1.36 (1.10, 1.69); **0.007** |
| **Quartile of ABSI** |  |  |  |
| Q1 | 1 (Ref) | 1 (Ref) | 1 (Ref) |
| Q2 | 1.26 (1.00, 1.59); **0.490** | 1.22 (0.96, 1.55); 0.100 | 1.16 (0.89, 1.51); 0.246 |
| Q3 | 1.73 (1.37, 2.17); **< 0.001** | 1.52 (1.18, 1.96); **0.002** | 1.37 (1.03, 1.83); **0.032** |
| Q4 | 2.05 (1.63, 2.56); **< 0.001** | 1.55 (1.19, 2.03); **0.002** | 1.36 (1.00, 1.86); **0.050** |
| *P* for trend | **< 0.001** | **0.001** | **0.037** |

Unadjusted model: non-adjusted model.

Adjust 1: Adjust for age, sex, race.

Adjust 2: Adjust for age, sex, race, body mass index, poverty income ratio, education levels, marital status, smoking status, alcohol consumption, hyperlipidemia, hypertension, diabetes mellitus and triglycerides.

* To facilitate data presentation and interpretation, ABSI values were multiplied by 100 due to their relatively small magnitude.

Abbreviations: ABSI, A Body Shape Index; CI, confidence interval.
