# Supplementary material for: Central obesity rather than BMI is associated with chronic pain: A cross-sectional analysis of NHANES
Source: PLoS One. 2025 Dec 4;20(12):e0337939. doi: 10.1371/journal.pone.0337939 (PMC12677471; doi:10.1371/journal.pone.0337939)
Supplement: S4 Table — Unadjusted model: non-adjusted model. Adjust 1: Adjust for age, sex, race. Adjust 2: Adjust for age, sex, race, body mass index, poverty income ratio, education levels, marital status, smoking status, alcohol consumption, hyperlipidemia, hypertension, diabetes mellitus and triglycerides. * To facilitate data presentation and interpretation, ABSI values were multiplied by 100 due to their relatively small magnitude. Abbreviations: ABSI, A Body Shape Index; CI, confidence interval. (DOCX) [file pone.0337939.s004.docx]

**Table S4*.*** Sensitivity analysis was conducted by redefining chronic pain as pain lasting more than one year.

| **Exposure** | **Unadjusted model** | **Adjust 1** | **Adjust 2** |
| --- | --- | --- | --- |
|  | Odds ratio (95% confidence interval) associated with chronic pain | | |
| **ABSI (continuous)*** | 1.72 (1.41, 2.10); **< 0.001** | 1.52 (1.25, 1.86); **< 0.001** | 1.38 (1.08, 1.76); **0.013** |
| **Quartile of ABSI** |  |  |  |
| Q1 | 1 (Ref) | 1 (Ref) | 1 (Ref) |
| Q2 | 1.56 (1.16, 2.08); **0.004** | 1.55 (1.17, 2.07); **0.003** | 1.49 (1.08, 2.05); **0.019** |
| Q3 | 1.52 (1.14, 2.02); **0.001** | 1.42 (1.09, 1.83); **0.010** | 1.30 (0.96, 1.76); 0.087 |
| Q4 | 2.17 (1.66, 2.83); **< 0.001** | 1.82 (1.35, 2.44); **< 0.001** | 1.60 (1.14, 2.24); **0.010** |
| *P* for trend | **< 0.001** | **< 0.001** | **0.021** |

Unadjusted model: non-adjusted model.

Adjust 1: Adjust for age, sex, race.

Adjust 2: Adjust for age, sex, race, body mass index, poverty income ratio, education levels, marital status, smoking status, alcohol consumption, hyperlipidemia, hypertension, diabetes mellitus and triglycerides.

* To facilitate data presentation and interpretation, ABSI values were multiplied by 100 due to their relatively small magnitude.

Abbreviations: ABSI, A Body Shape Index; CI, confidence interval.
