# Supplementary material for: Central obesity rather than BMI is associated with chronic pain: A cross-sectional analysis of NHANES
Source: PLoS One. 2025 Dec 4;20(12):e0337939. doi: 10.1371/journal.pone.0337939 (PMC12677471; doi:10.1371/journal.pone.0337939)
Supplement: S5 Table — Abbreviations: ABSI, A Body Shape Index. (DOCX) [file pone.0337939.s005.docx]

**Table S5*.*** Nonlinear *P*-values of ABSI and chronic pain at different knots.

| **Knots** | **Nonlinear *P*-values** | **Significance (*P* > 0.05)** |
| --- | --- | --- |
| 3 | 0.30081779 | Yes |
| 4 | 0.48291457 | Yes |
| 5 | 0.04580705 | No |
| 6 | 0.11850500 | Yes |
| 7 | 0.09584651 | Yes |
| 8 | 0.09654938 | Yes |

**Abbreviations**: ABSI, A Body Shape Index.
